# 

**Published:** 1908-12-05

**Authors:** 


### BOOKS RECEIVED.

T. Fisher Unwin.
"Vital Economy." By John H. Clarke, M.D.

University Tutorial Press.
"Physical Education and Hygiene." By W. P. Welpton, D.Sc.

Baillière, Tindall, and Cox.
"Handbook for Attendants on the Insane." Fifth edition.

Cassell and Co.
"The Diagnosis of Small-pox." By T. F. Ricketts, M.D., and J. B. Byles, M.B.

C. Griffin and Co., Ltd.
"Physiology and Pathology of the Urine." By J. Dixon Mann, M.D. Second edition.

THE

### GRADUATES' MEDICAL SCHOOLS.

DIARY FOR WEEK DEC. 7 TO DEC. 11.

**NATIONAL HOSPITAL FOR THE PARALYSED AND EPILEPTIC, Queen Sq., Bloomsbury, W.C.**

**At 3.30 p.m.**
**Dec. 8,** Dr. A. Turner, **Epilepsy.**
**Dec. 11,** Mr. Armour, **Surgery of the Nervous System.**

**NORTH-EAST LONDON POST-GRADUATE COLLEGE, Prince of Wales's General Hospital, Tottenham, N.**

**At 4.30 p.m.**

**Dec. 11,** Mr. Brooks, **Optic Neuritis.**

**ST. JOHN'S HOSPITAL FOR DISEASES OF THE SKIN, Leicester Square, W.C.**

**At 6 p.m.**
**Dec. 10,** Chesterfield Lectures, **Fungous Diseases of the Hair.**

**MOUNT VERNON HOSPITAL, FOR CONSUMPTION, Fitzroy Square, W.**

**At 5 p.m.**
**Dec. 10,** Dr. T. D. Lister, **Pain in the Chest.**

**MEDICAL GRADUATES' COLLEGE AND POLYCLINIC, 22 Chenies Street, W.C.**

**At 5.15 p.m.**
**Dec. 7,** Mr. Little, **Flat-foot.**
**Dec. 8,** Mr. Paton, **Optic Neuritis.**
**Dec. 9,** Dr. Ferrier, **The Pupil in Health and Disease.**
**Dec. 10,** Mr. Carless, **Some Points in the Surgery of the Biliary Passages.**

**LONDON SCHOOL OF CLINICAL MEDICINE, Seamen's Hospital, Greenwich, S.E.**

**At 2.15 p.m.**
**Dec. 7,** Sir Dyce Duckworth, **Dyspepsia due to Alcoholic Abuse.**
**At 3.15 p.m.**
**Dec. 10,** Sir Wm. Bennett.

**THE WEST-END HOSPITAL FOR NERVOUS DISEASES, Welbeck Street, W.**

**At 5 p.m.**
**Dec. 10,** Dr. Macnamara, **Syphilitic Affections of the Nervous System.**

**HOSPITAL FOR CONSUMPTION AND DISEASES OF THE CHEST, Brompton, S.W.**

**At 4 p.m.**
**Dec. 9,** Dr. Hector Mackenzie, **Cases from the Wards.**

**THE THROAT HOSPITAL, Golden Square, W.**

**At 5.30 p.m.**

**Dec. 7,** Mr. Parker, **Nasal Neuroses.**

**Dec. 10,** Mr. Rose, **New Growths of the Nose.**

**THE HOSPITAL FOR SICK CHILDREN, Great Ormond Street, W.C.**

**At 4 p.m.**
**Dec. 10,** Dr. Thursfield, **Pyelitis in Children.**

**THE POST-GRADUATE COLLEGE, West London Hospital, Hammersmith, W.**

**At 10 a.m.**
**Dec. 7 and 10,** Mr. Etherington Smith, **Surgical Demonstration.**
**At 12 noon.**
**Dec. 7,** Dr. Low, **Pathological Demonstration.**
**At 12.15 p.m.**
**Dec. 9,** Dr. Pritchard, **Practical Medicine.**
**At 5 p.m.**
**Dec. 7,** Mr. Lloyd, **Anæsthetics, II.**
**Dec. 8,** Dr. Low, **Bilharziosis.**
**Dec. 9,** Dr. Beddard, **Medicine, V.**
**Dec. 10,** Mr. Baldwin, **Surgery, V.**
**At 3 p.m.**
**Dec. 11,** Dr. Jones, **General Symptomatology of Insanity** (at Claybury Asylum, Woodford Bridge, Essex).

